# Dual gene expression cassette is superior than single gene cassette for enhancing sheath blight tolerance in transgenic rice

**DOI:** 10.1038/s41598-017-08180-x

**Published:** 2017-08-11

**Authors:** Subhasis Karmakar, Kutubuddin A. Molla, Kaushik Das, Sailendra Nath Sarkar, Swapan K. Datta, Karabi Datta

**Affiliations:** 1 Laboratory of Translational Research on Transgenic Crops, Department of Botany, University of Calcutta, 35 Ballygunge Circular Road, Kolkata, 700019 West Bengal India; 2 Crop Improvement Division, ICAR-National Rice Research Institute, Cuttack, 753006 Odisha India; 3 Visva Bharati University, Santiniketan, India

## Abstract

Sheath blight, caused by the necrotrophic fungal pathogen *Rhizoctonia solani*, is a serious and destructive disease of the rice. In order to improve sheath blight resistance, we developed three different kinds of transgenic rice lines. The first transgenic line overexpresses the rice *chitinase* gene (*OsCHI11*); the second contains the *Arabidopsis NPR1* (*AtNPR1*) gene and, the third has pyramided constructs with both the genes (*OsCHI11* and *AtNPR1*). This is a comparative study between the single-gene transgenic lines and the double gene transgenic in terms of their ability to activate the plant defense system. Rice plants of each individual construct were screened via PCR, Southern hybridization, activity assays, and expression analysis. The best transgenic lines of each construct were chosen for comparative study. The fold change in qRT-PCR and activity assays revealed that the pyramided transgenic rice plants show a significant upregulation of defense-related genes, *PR* genes, and antioxidant marker genes as compared to the single transgene. Simultaneous co-expression of both the genes was found to be more efficient in tolerating oxidative stress. In *R*. *solani* (RS) toxin assay, mycelial agar disc bioassay, and *in vivo* plant bioassay, pyramided transgenic plant lines were more competent at restricting the pathogen development and enhancing sheath blight tolerance as compared to single gene transformants.

## Introduction

Rice (*Oryza sativa* L.), being the most important staple food crop, is a major nutritional food supplement for more than half of the global population. One of the major constraints of rice productivity is the frequent occurrence of the diseases caused by fungi, bacteria, and viruses. The three major diseases that affect the rice crop are blast (causative agent: *Magnaporthe grisea*), sheath blight (causative agent: *Rhizoctonia solani*), and bacterial leaf blight (*Xanthomonas oryzae* pv. *Oryzae*)^1^. When considered in combination, these three diseases can account for an annual loss of as much as 50% of rice productivity^2^.

Sheath blight, caused by the fungus *Rhizoctonia solani* AGI-IA, is the second most devastating disease of rice^3^. This disease causes an annual yield loss of around 10–25%^4^. The damage caused by the sheath blight fungus includes a rapid decrease in chlorophyll content and the loss of photosynthetically active area. This occurs due to the development of lesions on both leaf blade as well as sheaths^5^. In addition, the array of lytic enzymes and toxins secreted by this necrotrophic fungal pathogen causes an alteration in the photosynthetic and respiratory processes of the green tissues^6^. The management of rice sheath blight is difficult due to the absence of the desired level of resistance in cultivated and wild rice. Resistance is conferred to rice solely by the presence of the non-race-specific resistance QTL factor; however, no *R* genes, corresponding to *R*. *solani* have been discovered to date^7^. Hence, it is imperative to develop alternative strategies for the development of durable and broad-spectrum resistance to sheath blight. Genetic engineering is considered as a promising alternative tool for enhancing the tolerance of rice plants toward sheath blight disease.

Systemic Acquired Resistance (SAR) is an innate plant defense strategy, where the expression of the defense genes increases; this enhances resistance toward pathogens^8^. SAR has been characterized in the model plant *Arabidopsis thaliana*, and it has been documented that the accumulation of pathogenesis-related (PR) proteins confers broad-spectrum protection against a wide variety of microorganisms^9^. *Arabidopsis NPR1* gene encodes a key SAR regulatory component^10^. The over-expression of *NPR1* in *Arabidopsis* showed that the NPR1 protein accumulates in the cytosol as an inactive multimeric protein complex^11^. The changes in the redox level during SA (Salicylic Acid) treatment or pathogen infection causes this protein complex to break into a monomeric form that is then transported to the nucleus where it interacts with the TGA family of basic leucine zipper transcription factors^12^. This interaction induces the expression of various *PR* genes^13^. In several crop plants such as tomato, tobacco, and rice^14–18^, *AtNPR1* or its orthologs have been found to be associated with the phenomenon of conferring enhanced resistance against a wide variety of pathogens. These observations strongly suggest that *AtNPR1* and its homologs could serve as important candidates for providing resistance against rice sheath blight.

Chitin, a ß-(1, 4) linked unit of amino sugar, constitutes about 3–60% of the cell wall in various fungi^19^. Chitinase catalyzes the hydrolysis of the ß-(1, 4) linkages of chitin. The inhibitory action of this enzyme is directly linked to a balloon-like swelling followed by the thinning of the growing hyphal tips leading to bursting of fungal hyphae. In addition, the fungal cell wall degradation products, which are generated in the process, especially the monomers and oligomers, act as potent elicitors of plant defense^20^. Among the various plant *chitinase* genes used in genetic transformation, rice *chitinases* have been most extensively studied. The resistance to a wide range of plant pathogenic fungi by the constitutive expression of rice *chitinase* gene has been well documented in various economically important plants such as rice^21^, grapevine^22^, and cucumber^23^. On the basis of aforementioned studies, the inhibition of chitin metabolism in fungi such as *R*. *solani*, via the expression of rice *chitinase*, is a promising strategy for controlling the disease like rice sheath blight.

In the present investigation, our objectives were to (i) generate rice plant lines harboring single as well as the pyramided construct of *OsCHI11* and *AtNPR1* genes under two different green tissue-specific promoters along with molecular evaluation of the transgenic plants; (ii) to identify and validate the best transgenic line for each construct; (iii) to compare single gene derived transgenic lines with the double gene construct derived transgenic lines using gene expression, histochemical, and biochemical analyses subsequent to the sheath blight infection; (iv) *in vitro* and whole plant bioassay of transgenic plants for measuring the tolerance level against sheath blight disease; and (v) to evaluate the agronomic and phenotypical parameters of different transgenic lines.

## Results

### Generation, molecular evaluation, and selection of best transgenic line of individual construct

**Figure 1:**
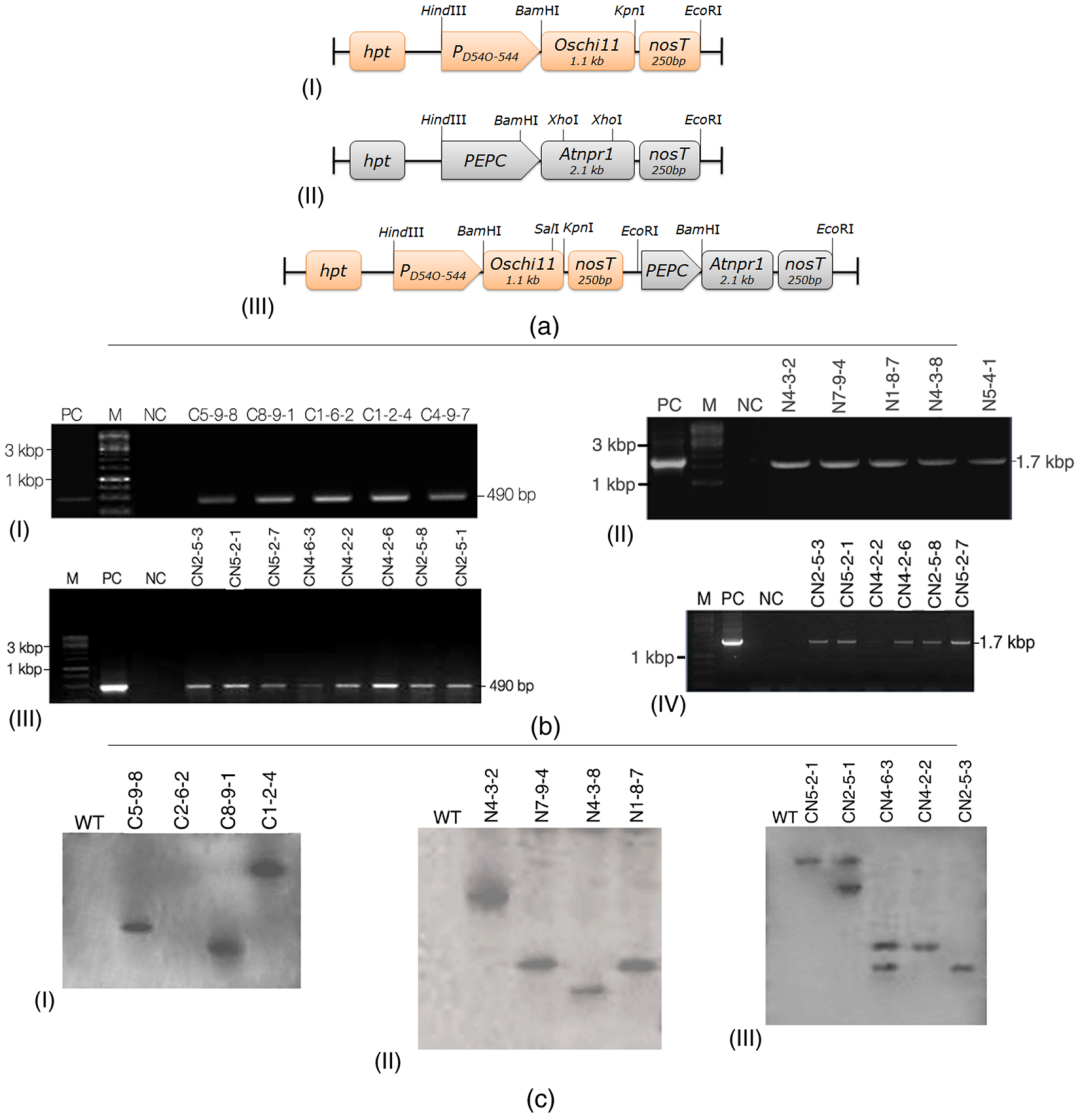
Diagrammatic representation of gene constructs and molecular evaluation of transgenic rice lines. (**a**) (I) Schematic representation of T-DNA construct harboring *OsCHI11* gene under the green tissue-specific promoter *P*_*D54O-544*_ used for rice transformation. (II) Diagrammatic representation of T-DNA construct *AtNPR1* gene placed under the control of maize green tissue-specific PEPC promoter. (III) Diagrammatic representation of the T-DNA construct used to transform mature embryogenic calli of jaldi-13 rice variety. *OsCHI11* and *AtNPR1* genes under the control of the rice green tissue-specific promoter (*P*_*D54O–544*_) and maize green tissue-specific promoter (*PEPC*), respectively. (**b**) (I) PCR analysis of T_2_ transgenic rice plants with partial gene-specific (*OsCHI11*) primers which amplified 490 bp product. (II) PCR analysis of T_2_ transgenic rice plants with partial gene-specific (*AtNPR1*) primers which amplified 1.7Kbp product. (III) PCR based screening of T_2_ transgenic and non-transformed WT performed with partial gene specific primer (*OsCHI11*) which amplified 490 bp product. (IV) PCR analysis performed with partial gene specific primer (*AtNPR1*) showing amplification of 1.7Kbp product. PC-Positive control and NC- Negative control. (**c**) (I) Southern blot analysis of T_2_ transgenic plants (C): genomic DNA digested with *Sal*I restriction enzyme and probed with 1.1 kbp *HPT* gene fragment. (II) Southern blot analysis T_2_ transgenic plants (N): genomic DNA digested by *EcoR*I restriction enzyme and probed with 1.1 kbp *HPT* gene fragment. (III) Southern hybridization analysis of T_2_ transgenic plants (C-N): genomic DNA was digested with *Sal*I restriction enzyme and probed with 1.1 kbp *HPT* (*hygromycin phosphotransferase*) gene fragment. WT represents wild type.

**Figure 2:**
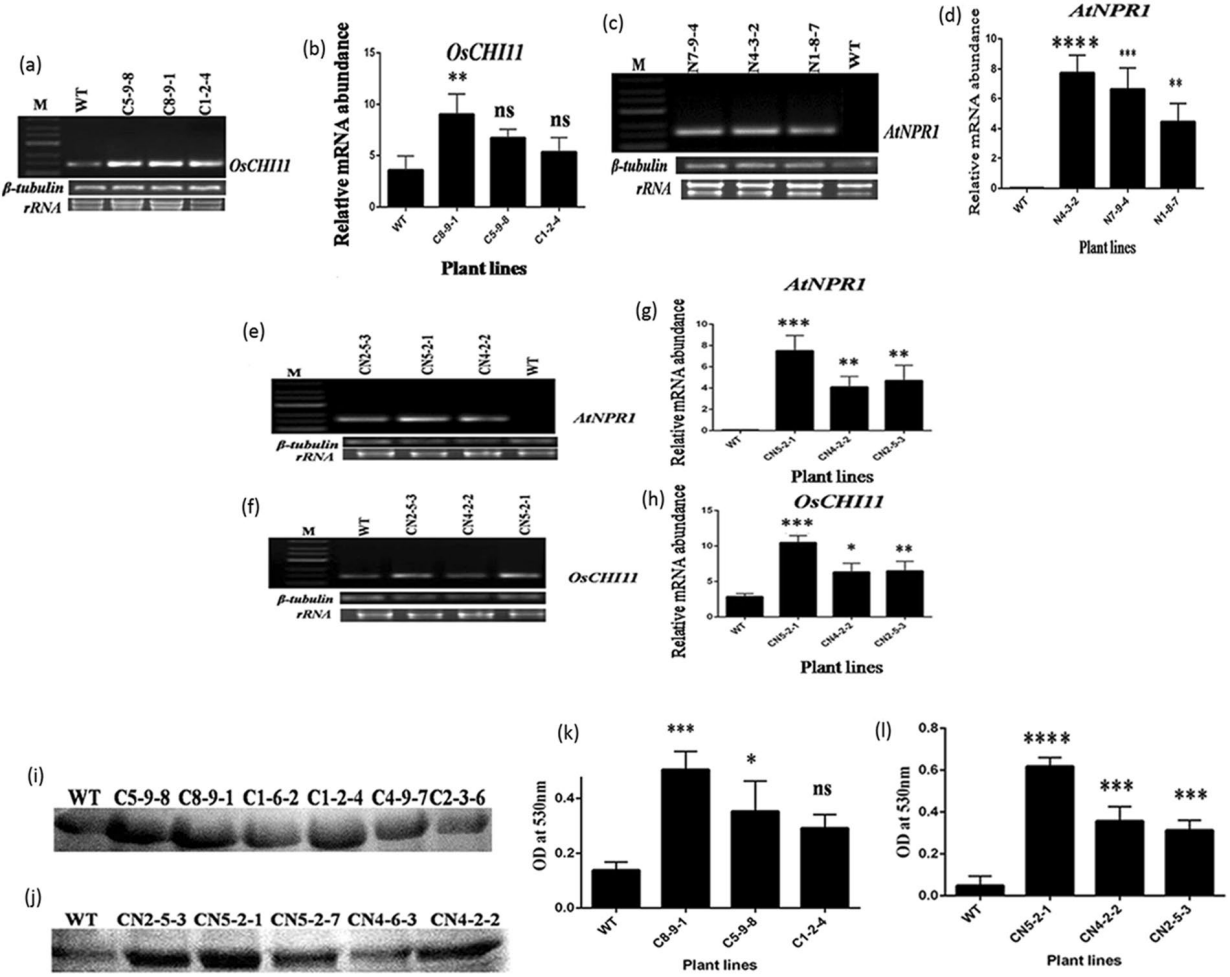
Expression analysis and activity assay of transgenic and wild type plants. (**a**) Semi quantitative RT-PCR analysis of selected T_2_ transgenic *OsCHI11* overexpressing rice lines using gene specific primers taking *β-tubulin* as reference control.(**b**) Relative quantity of rice *OsCHI11* mRNA in leaves of T_2_ (C8-9-1, C5-9-8, C1-2-4) overexpressing and WT rice lines as determined by RT-PCR (Real time PCR). (**c**) Semi quantitative RT-PCR analysis of selected T_2_ transgenic *AtNPR1* rice lines using gene-specific primers taking *β-tubulin* as internal control. (**d**) Relative quantity of *Arabidopsis NPR1* mRNA in leaves of T_2_ (N4-3-2, N7-9-4, C1-8-7) overexpressing and WT rice lines as determined by RT-PCR (Real time PCR). (**e**) Semi quantitative RT-PCR analysis of selected T_2_ transgenic *OsCHI11*-*AtNPR1* rice lines using gene-specific (*AtNPR1*) primer with *β-tubulin* as internal control. (**f**) Semi quantitative RT-PCR analysis of selected T_2_ transgenic *OsCHI11*-*AtNPR1* rice lines using gene-specific (*OsCHI11*) primer where *β-tubulin* acts as internal control. (**g**) Relative abundance of *AtNPR1* mRNA in T_2_ transgenic (CN5-2-1, CN4-2-2, CN2-5-3) pyramided lines and untransformed WT was determined by real-time PCR using SYBR green.(h) Relative quantity of *OsCHI11* mRNA in T_2_ transgenic (CN5-2-1, CN4-2-2, CN2-5-3) rice lines and non-transformed WT was determined real -time PCR using SYBR green. Values represent the mean ± SE of three independent experiments. (**i**) In-gel chitinase activity assay of T_2_ transgenic and non-transformed wild type (WT) plants. (**j**) In-gel chitinase activity assay of T_2_ pyramided transgenic and non-transformed wild type (WT) plants. (**k**) Activity assay (‘In-solution’) of chitinase in T_2_ transgenic and WT plants by dinitrosalycylic acid (DNSA) method. Activity was measured by spectrophotometer with an optical density of 530 nm (OD_530_). (**l**) ‘In-solution’ activity assay of chitinase in T_2_ transgenic and WT plants by dinitrosalycylic acid (DNSA) method. Chitinase activity was measured by spectrophotometer with an optical density of 530 nm (OD_530_). Values are presented as mean ± SE (n = 3).

Fifty-three independent T_0_ transgenic lines with a total of 140 plants were developed from *Agrobacterium-*mediated transformation with three different constructs. Hygromycin (50 mg/L) selected putative T_0_ plants were chosen to advance for T_1_ generation. On the basis of the phenotype, hygromycin response, and molecular characterization, ten plants containing each construct (i.e., CN, C, and N) independently were further advanced to T_2_ generation. PCR analysis of the T_2_ transgenic lines, using partial gene specific primers of *OsCHI11* and *AtNPR1* genes, revealed amplification of 490 bp and 1.7 kbp fragments, respectively, while no amplification was observed in non-transformed plants [Fig. 1b(I),(II),(III),(IV)]. The integration and transgene copy numbers were further examined by Southern hybridization and the transgenic lines containing a single copy of the transgene for each of the constructs were selected for further evaluation and comparison [Fig. 1c(I),(II),(III)]. The expression and transcript levels of the *OsCHI11* and *AtNPR1* genes were examined in different transgenic lines as also in non-transformed wild-type control (WT) at 2DPI (days post infection). The qRT-PCR results revealed that the expression levels of *OsCHI11* and *AtNPR1* genes increased substantially in the CN5-2-1 line (2.67 fold and 7.5 fold, respectively) with respect to the wild type control but the other two lines, i.e., CN4-2-2 (1.21 fold and 4.09 fold respectively) and CN2-5-3 (1.27 fold and 4.70 fold respectively), showed comparatively lower elevation (Fig. 2g and 2h). The differential expression in the transgenic and non-transformed plants were statistically significant (*F* = 25.60; *P* = 0.0002 for *OsCHI11* and *F* = 21.90; *P* = 0.0003 for *OsCHI11*). Similarly, as compared to WT, the maximum expression of *AtNPR1* gene was observed in N4-3-2 (7.76 fold) line whereas the lines N7-9-4 (6.62 fold) and N1-8-7 (4.46 fold) showed relatively lower expression (*F* = 28.55; *P* = 0.0001) (Fig. 2d). *OsCHI11* gene in the C8-9-1 (2.52 fold) line showed maximum elevation with respect to non-transformed WT but the other two transgenic lines, viz., C5-9-8 (1.87 fold) and C1-2-4 (1.49 fold), showed a comparatively lower expression (*F* = 7.700; *P* = 0.0096) (Fig. 2b).

With respect to chitinase activity, the C8-9-1 line was the best performer among the lines expressing the C-cassette, while the CN5-2-1 line showed the highest level of chitinase activity among the CN expressing lines (Fig. 2k and 2l). The difference between WT and transgenic lines was statistically significant (p < 0.005 in the case of C8-9-1 and p < 0.0001 in the case of CN5-2-1). A similar outcome was also obtained from the chitinase in-gel activity assay where the C8-9-1 and CN5-2-1 lines exhibited relatively strong lytic activity as compared to the other lines (Fig. 2i,j). The best T_2_ transgenic lines (C8-9-1, CN5-2-1, and N4-3-2) from each corresponding construct (C, CN, and N) were selected to validate the results further.

### Elevated expression of *PR* genes, SA and JA pathway genes after *R*. *solani* infection in transgenic lines

**Figure 3:**
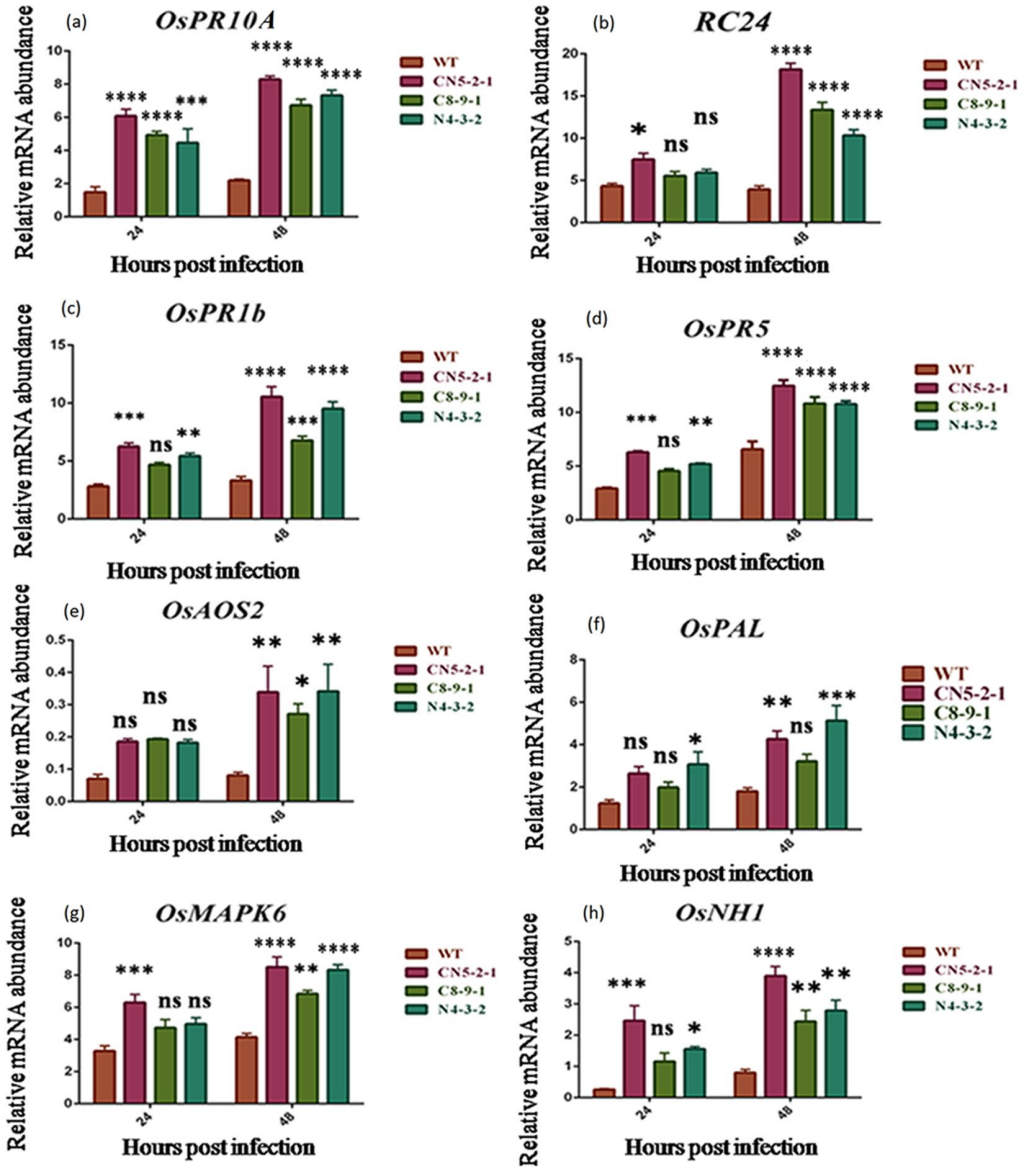
Real-time PCR analysis of some differentially expressed *PR*, SA and JA pathway genes in single transgene containing as well as pyramided rice lines. Total RNA isolated from leaves of both transgenic T_2_ and non-transformed WT plant, inoculated with sheath blight fungus *R*. *solani*, and harvested at 24 and 48 hpi (hours post infection). Experiment performed by SYBR green-based quantitative real-time PCR, using *β-tubulin* as internal control. Expression of (**a**) *PR10A*, (**b**) *RC24*, (**c**) *OsPR1b*, (**d**) *OsPR5*, (**e**) *OsAOS2*, (**f**) *OsPAL*, (**g**) *OsMAPK6 and* (**h**) *OsNH1 gene* in transgenic and wild type plants. Each bar represents the mean ± SE of three independent experiments.

In qRT-PCR analysis, the expression levels of different *PR* genes [*OsPR10a*, *OsPR1b*, *RC2*4 (*PR3*) and *OsPR5*], the markers for plant defense responses, were investigated in the CN5-2-1, C8-9-1 and N4-3-2 transgenic lines with respect to the non-transformed control at 24 and 48 h post-inoculation (Fig. 3a,b,c,d). The three JA and SA-dependent signalling pathway genes, phenylalanine ammonia-lyase (*OsPAL*), allene oxide synthase (*AOS*) and a rice homolog of *Arabidopsis NPRl* (*OsNHI*), along with a chitin-induced phytoalexins producing gene, *OsMAPK6*, were also investigated. The strongest induction of all these biotic stress marker genes, except for *OsPAL*, was observed in the CN5-2-1 transgenic line whereas the lines, N4-3-2 and C8-9-1 showed moderate and lower level of induction, respectively (Fig. 3e,f,g,h). The comparison between the different transgenic lines and WT at 24 and 48 h with respect to the relative fold change in qRT-PCR products for different biotic stress marker genes is given in Supplementary Table 3.

### Enhanced expression of different oxidative stress metabolizing enzymes upon *R*. *solani* infection in different transgenic lines

**Figure 4:**
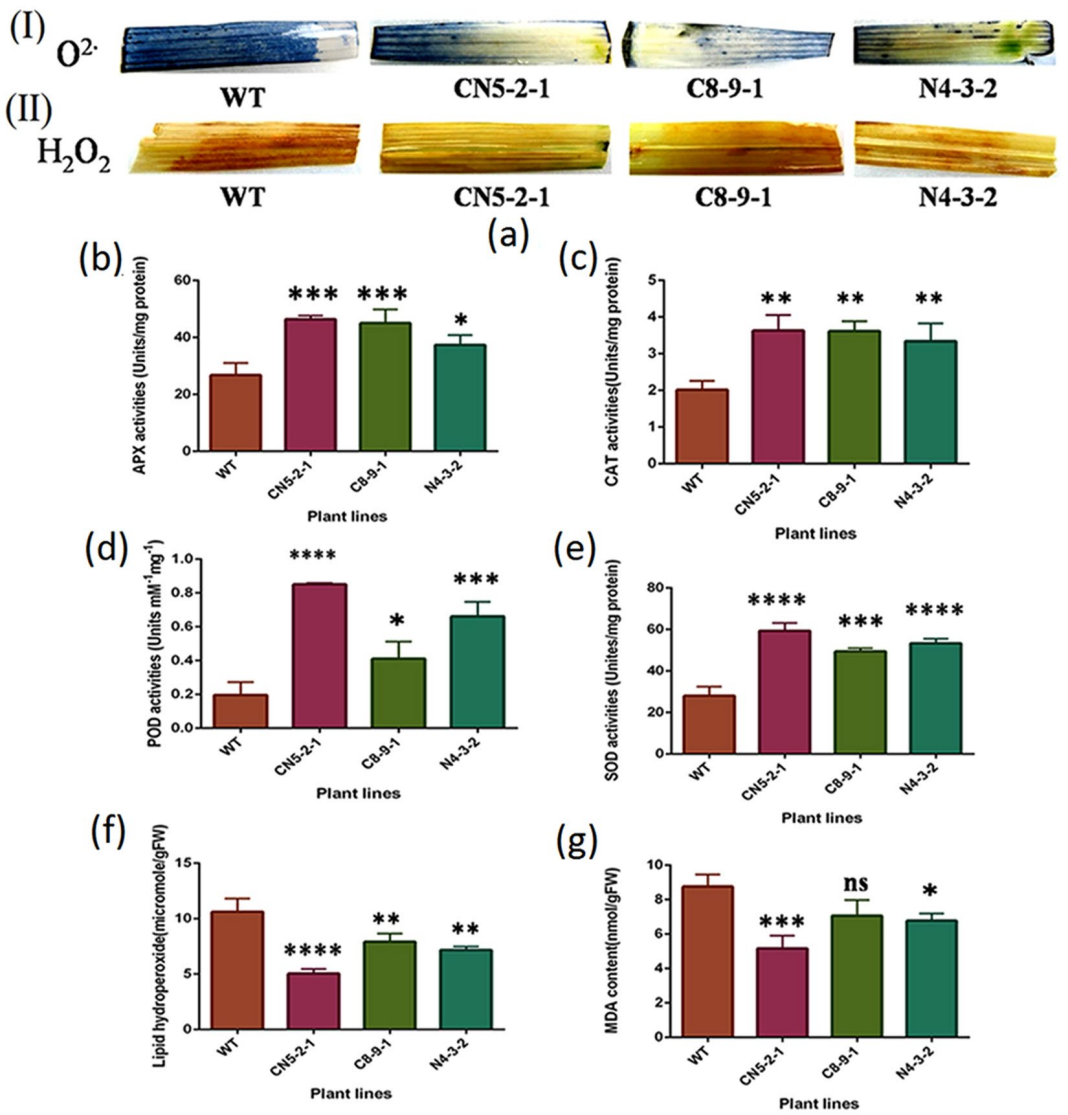
Oxidative damage and activities of different antioxidant enzymes in WT and T_2_ transgenic rice plants after sheath blight infection. (**a**) (I) *In situ* detection of O^2−^ by NBT staining in WT and T_2_ transgenic lines. (II) *In situ* detection of H_2_O_2_ by DAB staining in WT and T_2_ transgenic rice lines. (**b**) APX activity; (**c**) CAT activity; (**d**) POD activity; (**e**) SOD activity; (**f**) Determination of lipid hydroperoxide content in both transgenic and WT plants; (**g**) Determination of MDA accumulation in leaves of WT and T_2_ transgenic rice plant lines. Leaves were collected from transgenic and wild type (WT) plants after 2 days post infection (dpi). Data represent means ± SE calculated from three replicates. Three independent experiments performed.

Plants execute a complex antioxidative defense system in order to maintain a stable ROS-scavenging system for the maintenance of intracellular ROS homeostasis during stress conditions. Some antioxidant markers, such as SOD, POD, CAT, and APX activity levels, were studied in order to examine the defense responses and biochemical changes triggered by *R*. *solani* in the WT and transgenic lines. In contrast to WT, the pathogen inoculation significantly (*F* = 53.83; *P* < 0.0001) increased the activity of SOD in all three tested T_2_ transgenic lines: CN5-2-1, C8-9-1, and N4-3-2 lines (Fig. 4e). Similarly, CAT activity was elevated in all T_2_ transgenic lines, viz., CN5-2-1, C8-9-1, and N4-3-2 lines with respect to WT (*F* = 12.86; *P* = 0.0020) (Fig. 4c). The APX activity under stress increased significantly (*F* = 17.97; *P* = 0.0006,) in CN5-2-1, C8-9-1 and N4-3-2 lines when compared with non-transgenic plants (Fig. 4b). A substantial increase in POD activity was also observed in the transgenic lines CN5-2-1, C8-9-1 and N4-3-2 as compared to the infected WT plants (*F* = 42.15; *P* < 0.0001) (Fig. 4d). These results indicate that the expression of the corresponding transgene in transgenic rice plants is likely to enhance the activity of the antioxidant enzyme.

### Transgenic rice plants showed reduced ROS accumulation upon sheath blight infection

The two main sources of ROS, H_2_O_2_ and O_2_^−^, were detected in DAB and NBT staining, respectively, in a post-infection scenario. In DAB staining, two days after the inoculation, dark-brown polymeric oxidation products resulting from H_2_O_2_ were observed to accumulate in both WT as well as transgenic leaves; however, the leaves of WT exhibited significantly stronger staining as compared to the transgenic lines [Fig. 4a(II)]. Similarly, the blue polymerization product in NBT staining resulting from O_2_^−^ accumulation was observed to be substantially higher in WT in comparison to the transgenic lines [Fig. 4a(I)]. Among the transgenic lines, C8-9-1 exhibited the maximum accumulation of ROS and stained deeply with both NBT and DAB whereas the line CN5-2-1 was observed to accumulate the least amount of oxidation product.

Malondialdehyde (MDA), the final product of cellular membrane lipid peroxidation, is considered as a key parameter for measuring the extent of membrane damage in plants. The level of MDA in transgenic plants due to *R*. *solani* infection was found to be significantly lower in the CN5-2-1 and N4-3-2 lines as compared to WT at 2 DPI (Fig. 4g). However, in the case of the transgenic line C8-9-1, the difference in MDA content when compared to a non-transgenic control was not statistically significant. The accumulation of lipid hydroperoxide, another marker for membrane damage, was found to be significantly lower (*F* = 28.75; *P* = 0.0001) in the CN5-2-1, C8-9-1 and N4-3-2 transgenic lines at 2 DPI as compared to the WT (Fig. 4f).

### *AtNPR1-OsCHI11* pyramided line exhibits lower sheath blight symptom as compared to that of single-gene transgenic lines

**Figure 5:**
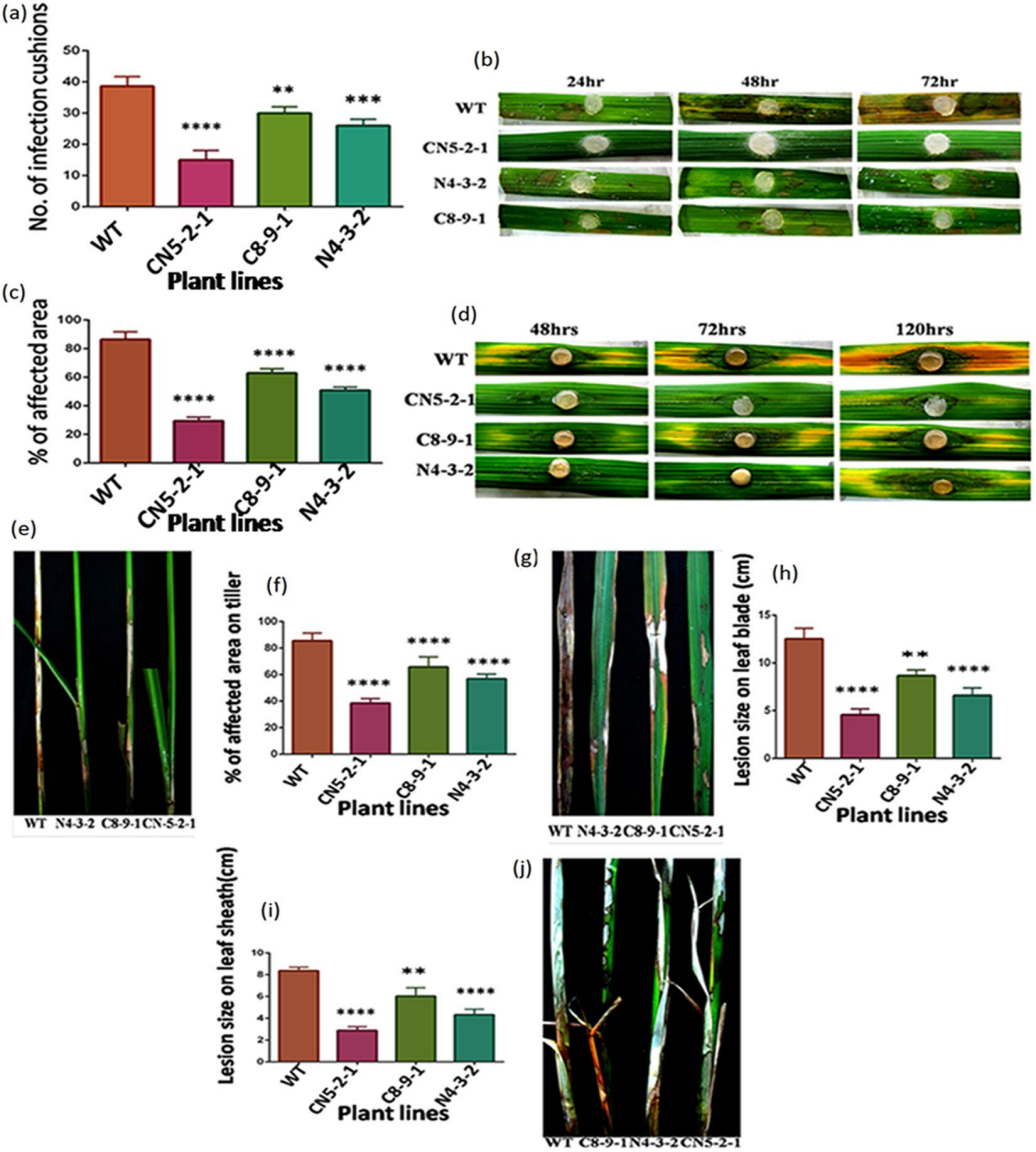
Evaluation of T_2_ transgenic lines against sheath blight disease along with non-transformed control through *In vitro* and *In vivo* plant bioassay. (**a**) Mycellial agar disc bioassay showing reduced infection cushion formation in T_2_ transgenic lines (CN5-2-1, N4-3-2, C8-9-1) than in the wild-type (WT). Experiments replicated three times. (**b**) Representative images of reduced lesion formation in transgenic leaves (CN5-2-1, N4-3-2, and C8-9-1) relative to WT in mycellial agar disc bioassay. (**c**) Bar diagram showing percentage of affected area after 72 hrs post infection in transgenic leaf samples than wild type (WT) in RS -toxin bioassay. (**d**) Images showing less affected area in transgenic leaves (CN5-2-1, C8-9-1, and N4-3-2) compared to WT control in the toxin bioassay. (**e**) Image showing reduced sheath blight symptoms development in rice tillers in T_2_ transgenic plants, than that in WT control after 21 dpi (Days post infection). (**f**) Percentage of infected area on tillers on T_2_ transgenic (CN5-2-1, C8-9-1, and N4-3-2) and WT plants after 21 dpi. (**g**) Images showing sheath blight symptoms on leaf blade of transgenic (CN5-2-1, C8-9-1, and N4-3-2) and non-transformed control. (**h**) Lesion size on leaf blade of WT and transgenic lines after 21 dpi. (**i**) Lesion size on leaf sheath of both WT and transgenic lines (CN5-2-1, C8-9-1, and N4-3-2) after 21 dpi. Values are presented as mean of 10 replicas ± SE. (**j**) Representative images showing reduction in symptoms development on rice sheath of transgenic lines as compared to WT after 21 dpi.

In order to evaluate the level of disease resistance of transgenic lines, we performed both *in vitro* and *in vivo* plant bioassay by infecting the plants with sheath blight fungus *R*. *solani*. In the detached leaf bioassay conducted using the *R*. *solani* mycelial agar disc, all transgenic lines (CN5-2-1, C8-9-1 and N4-3-2) showed significantly (*F* = 43.96; *P* < 0.0001) lower disease response when compared with that of wild-type control plant. After three days of inoculation, a stereomicroscope was used to assess disease severity by counting the number of infection cushions (served as an indicator of disease incidence) developed per leaf. The number of infection cushions in the CN5-2-1 line (15) was much lower than that those in the C8-9-1 (30) and N4-3-2 (26) lines; while the maximum number of cushions was found in the wild type (38.66) (Fig. 5a). The size of the lesions on the transgenic leaves was found to be less than on the control plant leaves; this also confirms an effective restriction of pathogen invasion (Fig. 5b). The non-transformed plants showed large yellow colored lesions, which subsequently turned brown after 72 h of the infection. The detached leaf bioassay, using partially purified *R*. *solani* toxin, revealed that the transgenic plant lines (CN5-2-1, C8-9-1 and N4-3-2) were able to significantly reduce (*F* = 141.3; *P* < 0.0001) the disease severity as can be deduced from the lower percentage of infected leaf area. After five days of incubation, it was noted that the transgenic plant CN5-2-1 line showed lowest percentage of affected area (29.51%) whereas C8-9-1 (62.91%) and N4-3-2 (50.84%) lines displayed moderate level of affec﻿ted area when compared to the WT control (86.43%) (Fig. 5c).

**Figure 6:**
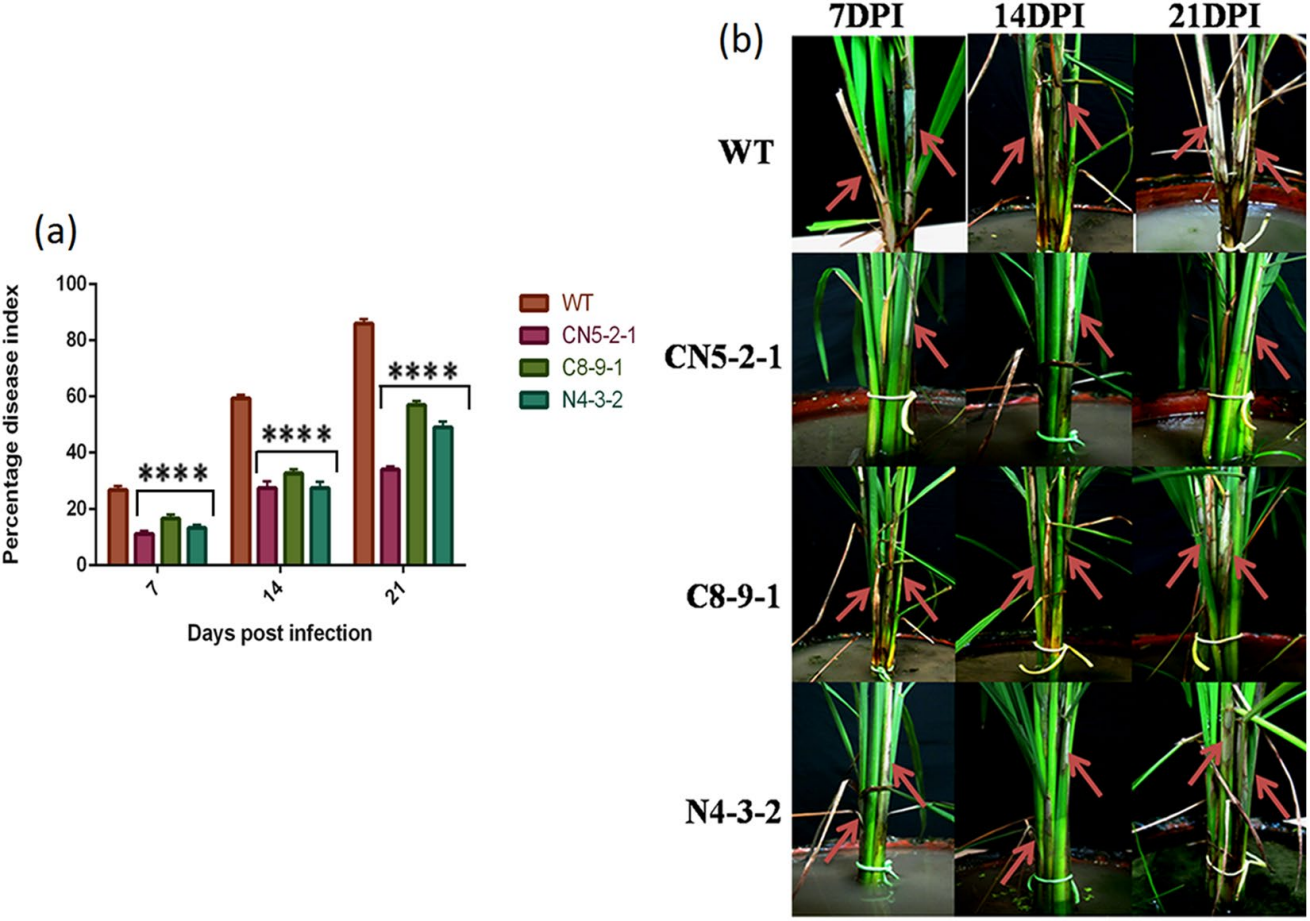
Whole plant bioassay of T_2_ transgenic and wild type (WT) plants with sheath blight fungus, *R*. *solani*. (**a**) Percent Disease Index (PDI) value in T_2_ transgenic plants with respect to wild type control at 7, 14 and 21 dpi (days post inoculation). The values represent as the mean ± SE (n = 15). (**b**) Representative images showing typical sheath blight symptoms development on rice tillers of T_2_ transgenic (CN5-2-1, C8-9-1, and N4-3-2) and non transformed wild type control. Pictures were taken at 7, 14 and 21 dpi. Red arrows indicate sheath blight symptoms.

The highly virulent *R*. *solani* AG1-1A (Hyderabad isolate) strain was used for whole plant bioassay of transgenic and wild-type control plants. When compared with the wild type control plants, the highest protection against sheath blight disease was exhibited by the CN5-2-1 transgenic plant line as demonstrated by the percent disease index (PDI), which was the lowest among the three transgenic lines. Prominent lesions appeared within 5–7 days on the wild type control as well as on transgenic plants. We assessed the disease severity by measuring the lesion size on the leaf blade (Fig. 5g) and leaf sheath (Fig. 5j), and by measuring the affected area on tillers (Fig. 5e) at 14 DPI (days post infection). The lesion size on leaf blade (Fig. 5h), sheath (Fig. 5i), and affected area on tillers (Fig. 5f) was observed to be smallest in the CN5-2-1 transgenic line (4.56, 2.89 and 38.53% respectively) as compared to WT control (12.53, 8.37 and 85.54%), whereas other transgenic lines C8-9-1 and N4-3-2 exhibited a moderate level of resistance. In all three cases, the mean differences between transgenic and non-transformed control plants were statistically significant (*P* < 0.0001). The mean disease scores of transgenic lines were significantly lower (*P* < 0.0001) than the wild-type control lines at 7, 14, and 21 days post-inoculation (DPI). At 7, 14 and 21 DPI, the CN5-2-1 line displayed the highest level of protection (PDI-11.18%, 27.55% and 34.03%, respectively) among the wild type control (PDI-26.88%, 59.35% and 86.04%) and other transgenic plant lines studied (Fig. 6a,b).

### Surviving tiller number and grain yield in transgenic plants after sheath blight infection

**Figure 7:**
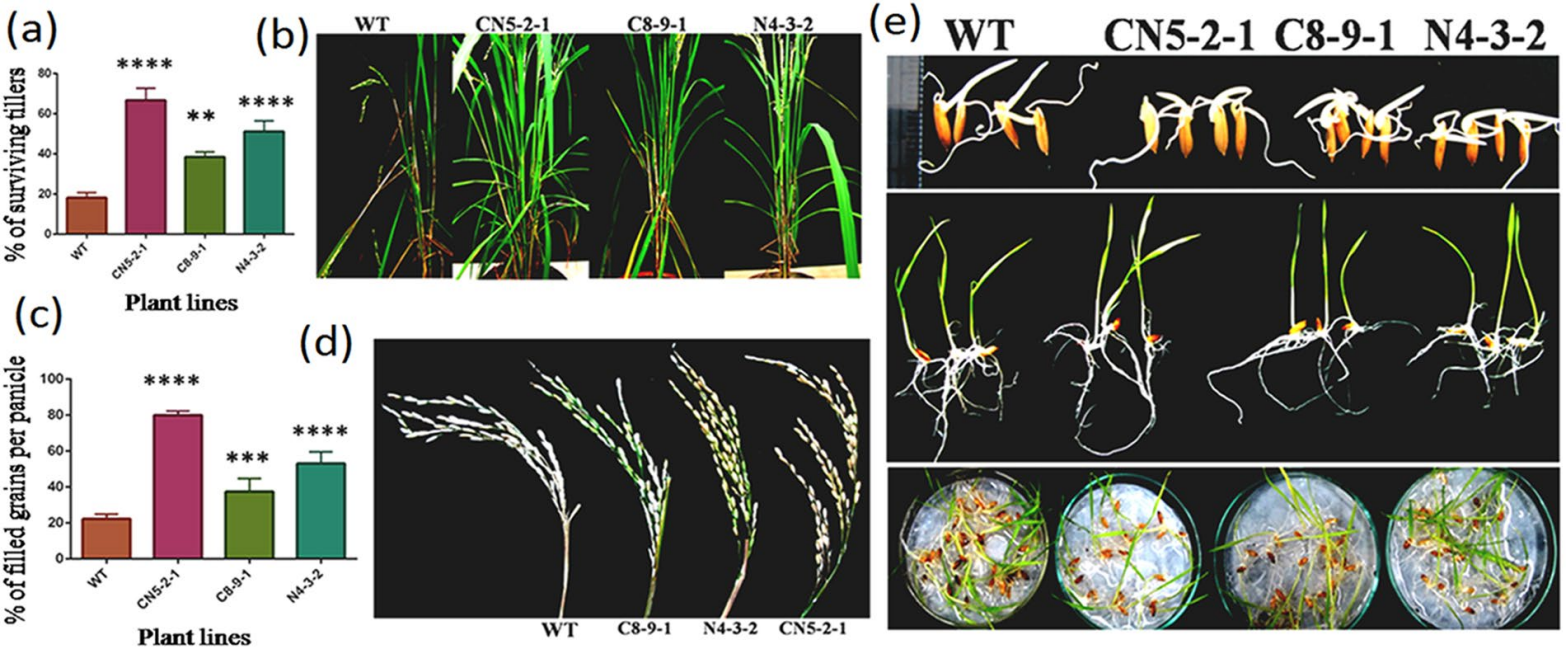
Enhanced resistance of transgenic lines to highly virulent isolates of *R*. *solani* and seed germination analysis. (**a**) Bar diagram showing percentage of survival tillers in T_2_ transgenic lines (CN5-2-1, C8-9-1, and N4-3-2) compared to wild type (WT) after 21 dpi. Bar represents mean ± SE of three independent experiments. (**b**) Images showing more number of surviving tillers in T_2_ transgenic lines (CN5-2-1, C8-9-1, and C4-3-2) compared to WT after 21 dpi. (**c**) Percentage of filled grains per panicle on transgenic and non-transformed control after 21 dpi. Bar represents mean ± SE of five independent experiments. (**d**) Images showing more grain count per panicle in transgenic plants after sheath blight infection with respect to WT at 21 dpi. (**e**) Images showing seed germination and phenotypic evaluation of T_3_ transgenic and wild type (WT) control seeds at juvenile stage.

In order to analyze the physiological and yield related components in transgenic and WT plants after sheath blight infection, the percentage of surviving fertile green tillers and the number of grains per panicle were measured at 30 DPI. The transgenic line CN5-2-1 demonstrated the maximum surviving green tillers (66.83%) as compared to the non-transformed control (18.25%) while the other two lines, C8-9-1 (38.54%) and N4-3-2 (51.21%), displayed comparatively lower tiller count than that of CN5-2-1 (*F* = 68.59, *P* < 0.0001) (Fig. 7a,b). Furthermore, all the three transgenic rice lines, i.e., CN5-2-1(80.04%), C8-9-1 (37.45%) and N4-3-2 (53.14%), exhibited significantly higher grain count per panicle [Fig. 7c] (F = 116, *P* < 0.0001) when compared to the non-transformed control (22.28%), which had the maximum number of unfilled panicles (Fig. 7d). These results clearly demonstrated that the transgenic rice lines provide a significant and durable protection against the sheath blight fungus and enhance the ability to overcome the damage caused by the infection.

### Transgene insertion does not alter seed germination or any other agronomically important traits in transgenic rice

The agronomic performance of all three transgenic plants was compared with that of the non-transgenic control. All transgenic and non-transformed plants were observed to grow normally; no significant difference was observed in terms of the number of tillers, plant height, and panicles (Table 1). In addition, the weight of 1000 dry seeds obtained from the transgenic plants was not significantly different from that obtained from the non-transgenic control. We also analyzed the seed germination potentials of T_3_ transgenic line and WT plants by performing the control germination test (CGT). The transgenic seeds, compared to the non-transformed controls, germinated normally without any morphological or phenotypic alterations (Fig. 7e).

**Table 1:** Agronomical data evaluation of transgenic lines.

Group	Plant height (cm)	Number of tillers	Panicle length (cm)	Panicle per plant	1000 seed weight (gm)
WT	55.43 ± 3.06	12.43 ± 1.99	16.44 ± 1.03	10.25 ± 0.60	19.22 ± 0.11
CN5-2-1	54.82 ± 5.33	17.32 ± 2.31	18.65 ± 1.11	12.09 ± 0.42	20.56 ± 0.12
C8-9-1	53.12 ± 3.33	16.83 ± 1.11	15.03 ± 2.87	11.09 ± 0.46	18.56 ± 0.19
N4-3-2	54.42. ± 4.90	13.83 ± 2.98	17.11 ± 2.00	10.10 ± 0.66	20.23 ± 0.17

All the essential parameters were measured for the transgenic plants with their wild type (WT) counterpart, grown in same condition in green house. The values represent the mean of progeny (T_3_) 10 plants from each line ± SE.

## Discussion

Sheath blight disease is one of the major threats to rice production. As the food security of approximately half of the world’s population is directly dependent upon rice, improving the resistance in this crop against sheath blight is imperative for sustainable rice production. Breeding for sheath blight resistance has not yet become a realistic option largely because of our inability to identify potent resistant germplasm, major resistant QTL or any other major resistance/defense-related gene. Thus improving host resistance by over-expressing different defense genes derived from rice and non-rice sources is an attractive alternate strategy for combating the pathogen. For developing host plant resistance, single defense gene expression and pyramiding of more than one gene are the common strategies. Pyramiding defense genes is considered superior to monogenic resistance in achieving durable resistance against pathogen concerned. Several studies have reported successful defense gene pyramiding in rice^24–27^. In the current study, we attempted to enhance sheath blight tolerance by developing transgenic rice lines expressing either the chitinase (*OsCHI11*) gene [Fig. 1a(I)] or *NPR1* (*AtNPR1*) [Fig. 1a(II)] gene alone and a line containing both the genes (*OsCHI11* and *AtNPR1*) [Fig. 1a(III)]. *Chitinase* is a well-known antifungal gene; its product confers improved resistance by breaking the fungal cell wall component chitin^28^. *Arabidopsis NPR1* is a master regulator of plant immunity that is known to provide broad spectrum resistance^29^. The expression of both the genes individually (*OsCHI11* and *AtNPR1*) in rice has been demonstrated to confer enhanced tolerance to sheath blight disease^17, 25, 30^. The primary objective of our study was to compare transgenic lines expressing either of single genes (*OsCHI11* or *AtNPR1*) with the line that contains both the genes. Three different types of transgenic lines were chosen based on the criteria that they should possess a single copy of their respective construct. C8-9-1 was selected for inclusion in the comparative study on the basis of *CHI11* gene expression (Fig. 2a,b) and chitinase enzyme activity (Fig. 2i,k), whereas N4-3-2 was selected on the basis of *AtNPR1* gene expression (Fig. 2c,d); the pyramid line CN5-2-1 was chosen on account of exhibiting the highest level of gene expression (Fig. 2e,f,g,h) as well as chitinase enzyme activity (Fig. 2j,l). A variety of parameters were used to compare these three lines so as to evaluate their efficacy in imparting tolerance towards the sheath blight pathogen *Rhizoctonia solani*.

The expressions of pathogenesis-related (*PR*) genes in response to invasion by pathogens are one of the most studied defense responses in case of plants^31^. In comparison to their wild-type counterparts, we observed an elevated level of expression of four different *PR* genes, viz., *PR1b*, *PR5*, *PR10a* and *RC24* in all three transgenic lines at 24 and 48 DPI (Fig. 3a,b,c,d). The expression of *PR1b* and *PR10a* was found to be higher in the *AtNPR1* expressed line as compared to the *CHI11* line while the reverse was true in the case of *RC24* expression. Earlier studies have also reported the induction of expression of *PR1b*, *PR10A* and *PR5* genes in *AtNPR1* transgenic rice plants upon pathogen infection^17, 32^. Upon infection, the over-expressed chitinase is hypothesized to act on the fungal cell wall to generate chitin oligomers which in turn intensifies the infection signaling and defense gene expression^33^. In all cases, the pyramided line CN5-2-1 was observed to exhibit a faster and stronger activation of endogenous *PR* genes in response to *R*. *solani* infection as compared to N4-3-2 and C8-9-1. The stronger activation of these defense genes in the pyramided line is likely to be an outcome of the synergistic effect of NPR1 and chitinase. The expressions of four other endogenous defense-related genes, *viz*., *PAL*, *AOS2*, *NH1*, *MAPK6* were also found to be more enhanced upon pathogen infection in transgenic lines than that in their wild-type counterpart. Among the transgenic lines, the pyramided line showed the highest accumulation of transcripts of all the above-mentioned genes except for *PAL* (Fig. 3e,f,g,h). The expression of the *AOS2* gene, a key enzyme in JA biosynthetic pathway, was also observed to be induced upon fungal infection. The over-expression of this gene has been linked to an enhanced *PR* gene expression and increased pathogen resistance^34^. *R*. *solani* is a necrotrophic fungus and it has been well established that JA-dependent defense pathways are conventionally activated in response of necrotroph^35^. Fungal MAMPs (microbial-associated molecular patterns), such as chitin elicitor, have been found to activate *OsMPK6* so as to produce phytoalexin momilactone and phytocassane^36^. In our study, we found significant up-regulation of the *OsMPK6* gene at 48 dpi. The transcripts of the *PAL* genes, which are related to the SAR pathway induction in the plant, were found to be accumulated more in N4-3-2 than the other two lines. Our result is in concurrence with an earlier study where PAL expression was reported to be enhanced in transgenic rice in response to *R*. *solani* infection^3^.

An increased accumulation of reactive oxygen species (ROS) is a common phenomenon in stress-related conditions^37^. The elevated activity of antioxidant enzymes is one of the major means by which plants curtail ROS-related damage. For neutralizing superoxide (O_2_^·−^) and hydrogen peroxide (H_2_O_2_), SOD, POD, CAT and APX are the key enzymes responsible. SOD acts as the first line of defense against membrane lipid peroxidation. It dismutases the superoxide radical into less damaging species like O_2_ or H_2_O_2_. POD, CAT and APX act as the next line of defense by detoxifying peroxides into H_2_O. In the present study, the activity of these antioxidant enzymes was observed to be induced upon *R*. *solani* inoculation (Fig. 4a,b,c and d). The antioxidant enzyme activities were similar or higher in the pyramided line compared to the other two lines after sheath blight infection. Our results are in accordance with a previous study which reported higher SOD and POD activity levels in hybrid rice plants infected with *R*. *solani* ^38^. Another study has also presented data that support a significant increase in SOD, CAT, APX, and POD activity in rice leaf sheath in response to *R*. *solani* infection^39^. Lignin or suberin mediated physical barrier formation also plays an important role in limiting pathogen invasion. Peroxidase enzyme helps in cross-linking lignin monomers and increasing plant cell wall rigidity^40^. As evidenced from DAB and NBT staining, transgenic plants showed less accumulation of ROS than the wild type plants. The pyramided line performed better in terms of lower accumulation of ROS as compared to the other two lines [Fig. 4a(I),(II)]. This result could be correlated with the higher activity of antioxidant enzymes in the pyramided line. Malondialdehyde (MDA) and lipid hydroperoxide are the two products generated by lipid peroxidation in plants. These two products are commonly correlated with the level of membrane damage caused by peroxidation. Interestingly, the pyramided line showed the lowest level of MDA and lipid hydroperoxide accumulation compared with the other two lines as well as wild type control (Fig. 4f,g). These observation concords well with the antioxidant enzyme activity and ROS accumulation data, as higher enzyme activity and lower ROS accumulation, can be directly correlated with lesser MDA and lipid hydroperoxide formation. This result indicates that the pyramided line that co-expresses both genes is better equipped than transgenic lines expressing either *AtNPR1* or *OsCHI11* to cope with the oxidative stress generated due to pathogen infection. It also has more potential to maintain homeostasis than the two lines studied.

As the next step toward evaluating the three transgenic lines, whether the elevated levels of defense gene expression and ROS homeostasis in the transgenic lines are capable of being translated into increased sheath blight disease resistance was studied. Three independent *R*. *solani* infection experiments, two detached leaf bioassays (Fig. 5b,d), and one whole plant bioassay (Fig. 6b) were performed on transgenic plants as well as on the wild-type control plants in order to assess their tolerance level. The bioassays using mycelial agar discs revealed that compared to the wild type, there was a significant reduction in the number of infection cushions in all transgenic lines, where the pyramided line (CN5-2-1) showed the highest level (61.2%) of reduction. Infection cushions develop from the aggregation of convoluted hyphae, which play an important role in direct cuticular penetration^3^. Therefore, a reduction in the number of infection cushions can be directly co-related to the level of tolerance. In a similar fashion, when RS toxin was applied on pricked leaves of transgenic plants, the pyramided line displayed smallest area of necrosis in contrast to other lines. However, the *NPR1* expressing line performed better than the *CHI11* expressing line in both the detached leaf bioassays. RS toxin, a host-specific toxin secreted by *R*. *solani*, is capable of producing all symptoms of the sheath blight disease^41^. Sheath blight sensitivity of rice cultivars has been demonstrated to be well correlated with the sensitivity to phytotoxin from *R*. *solani* ^42^. In the same way, whole plant bioassay comprising of analysis of percent disease index, lesion size on leaves, lesion size on sheath and affected area on tiller, established that the pyramided line had a stronger and more durable tolerance toward sheath blight as compared to the single-gene transgenic line (Figs 5g,j,e and 6a). The superior performance of the pyramided line can be attributed to the faster and stronger activation of rice defense genes during pathogen infection. The combination of *NPR1* and *chitinase* in pyramided line was able to instigate the plant defense system to a higher level than either gene could do alone. This fact can also be visualized by the stronger activation of *PR* genes and other defense-related genes in the pyramided line as compared to the single-gene transgenic lines. An additional contribution to the increased sheath blight tolerance of transgenic lines may derive from the higher expression of *MAPK6*, which might have contributed to increased accumulation of rice phytoalexins^36^.

The constitutive expression of defense genes, especially those associated with the regulatory systems, for induced resistance may lead to different types of phenotypic shortcomings^43^. The expression of *AtNPR1* that is under the control of a constitutive promoter has been shown to affect adversely rice plant phenotype^32, 44^. Although pyramiding of alfalfa *β-1*,*3-glucanase* gene (*AGLU1*) and rice *chitinase* gene (*RCH10*) under the control of constitutive promoter conferred tolerance to *R*. *solani*, but it had a negative impact on seed germination^45^. Recently, it has been demonstrated that the tissue-specific expression of *AtNPR1* can bypass phenotypic costs while imparting tolerance to sheath blight^17^. In the present study, two genes of interest under the control of two different green tissue-specific promoters, viz., *P*_*D54O-544*_^3^ and *P*_*PEPC*_^25^ have been over-expressed in order to avoid any possible phenotypic cost. The analysis of several phenotypic and morphological parameters revealed no significant phenotypic abnormalities in any of the three different transgenic lines (Fig. 7e).

The current study indicates that the synergistic effect of *AtNPR1* and *OsCHI11* genes in transgenic rice plants leads to a better and improved performance against sheath blight than that of a single individual gene. It is evident from this study that combining a regulatory gene (*NPR1*) with a functional *PR* gene (*CHI11*) leads to the formation of a synergistic effect in rice with superior sheath blight tolerance properties. Stronger defense gene activation, higher activity of antioxidative enzymes, lower ROS accumulation and lesser membrane peroxidation account for the superior performance of pyramided line against sheath blight tolerance. However, for a better understanding, global transcriptome analysis of the transgenic lines is imperative. The strategy used in this study has the potential to be extrapolated to other economically important crop species for improving tolerance against fungal infection levels.

## Methods

### Cloning and vector construction

The maize *PEPC* (1.2 kb) (Ac no: x15642.1) promoter was taken out from a *pUC19*-*P*_*PEPC*_ *-OsOXO4-NOS* vector^25^ by *Hind*III*-Bam*HI digestion and inserted at the MCS of *pCAMBIA1301* (*Gus* free)^46^, to generate *pCAMBIA1301-P*_*PEPC*_. *NPR1-NOS* fragment was released from *pBI-PNN* plasmid^17^ construct by digestion with *Bam*HI*-Eco*RI and sub-cloned downstream of *P*_*PEPC*_ in the *pCAMBIA1301-PEPC* vector to generate *pCAMBIA1301-P*_*PEPC*_ *-NPR1-NOS*. This vector construct is designated as N.

Another *OsCHI11* gene construct was liberated from the vector *pUC19-P*_*D54*O*–544*_-*OsCHI11-NOS* ^25^ by *Hind*III*-Eco*RI digestion and inserted at the MCS of *pCAMBIA1301* so as to generate *pCAMBIA1301-P*_*D54O–544*_ *-O*s*CHI11-NOS*. The name of this vector construct is abbreviated as C.

The vector *pUC19*-*P*_*PEPC*_ *-OsOXO4-NOS* ^25^ was digested by *Bam*HI-*Eco*RI to eliminate the *OsOXO4-NOS* fragment and an *NPR1-NOS* fragment released from the *pBI-PNN* ^17^ plasmid was subcloned downstream of *pUC19*-*P*_*PEPC*_ to generate *pUC19*-*P*_*PEPC*_ *-NPR1-NOS*. This construct was again digested with *Eco*RI and the cassette was inserted into the *Eco*RI site of *pCAMBIA1301-P*_*D54O–544*_ *-O*s*CHI11-NOS* in order to generate *pCAMBIA1301-P*_*D54O–544*_ *-O*s*CHI11-NOS- P*_*PEPC*_ *-NPR1-NOS*. This construct is abbreviated as C-N.

### Rice transformation and analysis of transgenic plants

Rice (*Oryza sativa* L. subspecies *indica* cv. Jaldi-13) was transformed through the *Agrobacterium*-mediated method^30^ using 20–25 days old embryogenic calli as explants. The transformants were selected on Murashige-Skoog plates^47^ supplemented with 50 mg/L hygromycin B. The plants were grown in a growth chamber. PCR screening was performed using partial gene-specific primers for the *OsCHI11* and *AtNPR1* genes (Supplementary Table 1).

### Southern hybridization

Total genomic DNA was extracted from greenhouse grown T_2_ transgenics as well as WT rice plants as per the methodology previously described^48^. Southern blot analysis was performed as per standard procedures^49^. A minimum of 15 µg of genomic DNA was digested with *Sal*I (C and C-N constructs) or *Eco*RI (N construct) by incubating the reactions overnight at 37 °C. Digested DNA was electrophoresed on a 1% agarose gel at 50 V. The gel was denatured, neutralized, and blotted onto nylon membranes (Amersham Pharmacia, USA). Southern blots were hybridized with the partial *HPT* (*hygromycin phosphotransferase*) gene fragment, which acted as the probe. This gene fragment was labeled with (α-3^2^P) dCTP using a Deca LabelTMDNA Labeling Kit (Roche) as per standard procedures prescribed by the manufacturer.

### RNA isolation and semiquantitative and quantitative reverse transcription (qRT)-PCR

Total RNA was isolated from 100 mg of leaf tissues using a commercially available RNeasy Plant Mini Kit (Qiagen, USA). This was followed by DNaseI (Fermentas, Canada) treatment. Of RNA, 5 µg was reverse-transcribed into cDNA using a Maxima First Strand cDNA Synthesis Kit (Fermentas, Canada) as per the manufacturer’s instructions. qRT-PCR was performed in CFX-96 real-time PCR system (Bio-Rad) using a SYBR Green Kit (Fermentas, Canada). Rice *β-tubulin* gene was used as an endogenous control for qRT-PCR. A list of primers used in the procedure is given in Supplementary Table 2. Quantitative variation was evaluated by the 2^−ΔΔ*C*T^ method^50^. The specificity of PCR amplification was verified using a two-pronged strategy: analysis with a heat dissociation curve (65–95 °C) after the PCR procedure as well as by running the amplified products on a 2% agarose gel.

Each experiment was performed in triplicates using three independent tissue samples. Semi-quantitative RT-PCR was performed using 1 µL of cDNA as template. High fidelity Taq polymerase (Fermentas, Canada) was used in the procedure.

### Chitinase activity assay

In-solution chitinase activity was determined according to a previously described protocol^51^ with modifications. The preparation of colloidal chitin has been described earlier^52^. The incubation of colloidal chitin with total protein was performed in a water bath at 45 °C for 1 h. HCl (100 µL, 1 N) was used to terminate the reaction. After 10 min of incubation on ice, the mixture was centrifuged at 13,000 rpm for 10 min in order to precipitate undigested substrate. The amount of reducing sugar released was measured in a spectrophotometer at 530 nm using dinitrosalicylic acid (DNS) reagent^53^. ‘In-gel’ activity assay for chitinase was performed as previously described in literature^54^. Total protein profile was resolved on a 12% discontinuous SDS-PAGE containing 0.05% (w/v) colloidal chitin. After electrophoresis, the gel was transferred into a 200 mM sodium acetate buffer (pH 5) solution containing 1% (v/v) deionized Triton X-100 and incubated in a shaking water bath for 2 h at 37 °C. The gel was stained for 5 min with Calcofluor white M-2R (0.01%) (Fluka, Sigma, USA) dissolved in 50 mM Tris-HCl (pH 8.0) and then washed several times with distilled water. Lytic zones were observed and photographed using a gel documentation system (BioRad, Hercules, California, USA).

### Histochemical staining

The generation of superoxide (O_2_^−^) radicals and hydrogen peroxide (H_2_O_2_) after sheath blight infection was analyzed through histochemical staining^55^. For O_2_^−^ detection, the leaves from both infected wild type and transgenic plants were floated in 50 mM sodium phosphate buffer (pH7.5) containing 0.2% fresh NBT solution at ambient temperature until the appearance of insoluble dark blue color. For detection of H_2_O_2_, the leaves were immersed in fresh DAB solution (1 mg mL^−1^ pH 3.8) prepared in 10 mM phosphate buffer (pH 7.8), and incubated overnight in light until brown spots were observed. The stained leaves were bleached in 95% ethanol in a boiling water bath and stored in 70% ethanol.

### Activity assays of different reactive oxygen species (ROS) scavenging enzymes

The activity assays for various antioxidant enzymes were performed using total protein obtained from leaf samples (WT and transgenic) that were collected at different time intervals after post-infection^39^.

#### Superoxide dismutase

The SOD activity assay was performed as per a method previously described^56^. The inhibition of the photochemical reduction of NBT was measured at 530 nm in a spectrophotometer. One unit of SOD activity was defined as the amount of enzyme required to cause 50% inhibition of the reduction of NBT.

#### Ascorbate peroxidase (APX)

APX activity was measured as previously described^57^. The decrease in absorbance was recorded at 290 nm for 180 seconds. Enzyme activity is expressed as units per milligram of protein. Calculation was done using the extinction coefficient of Ascorbate (E): 2.8 mM^−1^cm^−1^.

#### Catalase (CAT)

CAT activity was measured following the procedure described previously^58^. Enzyme activity was estimated by monitoring the decrease in absorbance of H_2_O_2_ at 240 nm for 180 seconds. Activity is expressed as units per milligram of protein.

#### Peroxidase (POD)

Peroxidase (POD) activity was measured using the protocol designed^59^ with modifications. The increase in absorbance at 470 nm was recorded within 120 seconds after addition of enzyme extract; enzyme activity is expressed as mM H_2_O_2_ decomposed per milliliter of total soluble protein.

### Lipid peroxidation assay

Lipid peroxidation was estimated by determining the MDA (malondialdehyde) content as previously described^60^ with some modifications. The MDA concentration was calculated using the extinction coefficient 155 mM^−1^ cm^−1^ and is expressed as nmole/g.

### Lipid hydroperoxide assay by ferrous xylenol orange

Lipid hydroperoxide content was spectrophotometrically determined using the xylenol orange assay^61^. Absorbance was recorded at 560 nm and the values are expressed as H_2_O_2_ equivalent µmol/g.

## Bioassay

### Detached leaf bioassay with mycelial agar disc

Detached leaf bioassay using mycelial agar disc was performed in accordance with previous report^3, 17^. Sterilized Petri plates were lined with sterile cotton pads and sterile distilled water was sprayed to maintain moisture. Detached leaves, after surface sterilization, were placed on sterile glass slides so that both ends were inserted into the slits of moistened Whatman 3 mm filter paper. A mycelial block (5 mm in diameter), grown on a 5 day old PDA culture of *R*. *solani*, was placed on the middle of the leaf surface. The lid was sealed with parafilm and kept at 28 °C for 3 days.

### RS-toxin isolation and detached leaf toxin bioassay

RS-toxin isolation and bioassay were done following previously described method^41^. Briefly, 50 μL toxin solution was used to inoculate the plant material. An injury was inflicted at the middle of each detached leaf piece with the help of a sterile needle. The filter paper disks of 5 mm diameter, soaked with the toxin sample, were placed on the injured leaf section. This set up was incubated at 28 °C (12 h of light and 12 h of darkness) for 5 days. After 5 days, symptom development was assessed.

### Bioassay with whole plant

Whole plant bioassay of transgenic and WT plants were performed as described previously^62^. The rice hull-rice grain inoculum (1 g) of *R*. *solani* was placed in between the stem and the basal leaf sheath of each tiller at about 3 to 4 cm above the water line and tied with rubber bands. Water was sprayed every morning so as to maintain a moistened environment. Disease assessment was done 7, 14 and 21 days after infection; a scale of 0–9 as described in Standard Evaluation System for Rice (IRRI 2002) was used for the purpose.

### Seed germination assay

The germination capacity of T_3_ transgenic seeds was compared to their non-transgenic counterpart using the controlled germination test (CGT) as described previously^63^. Seeds were soaked in sterile distilled water for 8 h at 30 °C, and transferred to fresh water (CGT) for an additional 12 h. After incubation, the seeds were rinsed two to three times in sterile distilled water and were germinated on Whatman filter paper soaked with distilled water and kept at 30 °C in a growth chamber.

### Agronomic evaluation of transgenic plants

Different agronomic parameters such as plant height (cm), panicle length (cm), the number of effective tillers, number of panicles per plant (PPP) and dry weight of 1000 grains were recorded for transgenic as well as non-transgenic wild type plants. Ten randomly chosen plants from each transgenic line grown under greenhouse conditions were considered for evaluation.

### Statistical analysis

All statistical analyses were conducted using the GraphPad Prism 6 software. The data were represented as the mean value derived from three independent experiments wherein each experiment was done in triplicate. The results are represented as means ± standard error (SE). Additionally, the differences between mean were analyzed by the Bonferroni Post-tests.

## Electronic supplementary material

Supplementary information
