# Supplementary material for: Dual gene expression cassette is superior than single gene cassette for enhancing sheath blight tolerance in transgenic rice
Source: Sci Rep. 2017 Aug 11;7:7900. doi: 10.1038/s41598-017-08180-x (PMC5554252; doi:10.1038/s41598-017-08180-x)
Supplement: Supplementary file 1 — Supplementary information [file 41598_2017_8180_MOESM1_ESM.pdf]

Dual gene expression cassette is superior than single gene cassette for enhancing sheath blight tolerance in transgenic rice

Subhasis Karmakar[1], Kutubuddin A. Molla[1,2], Kaushik Das[1], Sailendra Nath Sarkar[1] ,Swapan K. Datta[1,4]& Karabi Datta[1],*

*(Corresponding author)

Supplementary Table.1 List of primers used in PCR screening of transgenic plants

Gene	Primer sequence (from 5'- 3')	Amplicon size	Annealing temperature
OsCHI11	F-TCGCCTCCATCATATCGCCCTC<br>R-CGTCATCCAGAACCAGAACGCC	490bp	53°C
AtNPR1	F-ATGGACACCACCATTGATGG<br>R-TCACCGACGACGATGAGAGAG	1.7 kbp	58.1°C
HPT	F-CGCCGATGGTTTCTACAAAGA<br>R-TCAATGACCGCTGTTATGCG	495 bp	58.6°C
NOS	F-GGTACCGATCGTTCAAACATTTGGC<br>R-GAATTCCCGATCTAGTAAC	260 bp	50°C

Supplementary Table. 2 Primers for quantitative real-time polymerase chain reaction (qRT-PCR).

Gene	Primer sequence (5'-3')	Accession number	Amplicon size (bp)	Tm of amplicon
OsCHI11	F-GTTCTGGTTCTGGATGACGC<br>R- GCCGTTGATGATGTTGGTGA	X54367	139	87.50
AtNPR1	F- CATTCTCTCAAAGGCCGACT<br>R-AAGACGTTGAGCAAGTGCAA	NM_105102	159	80.00
OsPAL/ZB8	F-GTGTTCTGCGAGGTGATGAA<br>R-TTCCCTCCAAGATGTGCTTC	X87946.1	118	81.00
OsNH1	F-TGAAAGAAGGGACCCACAAC<br>R- AGGTGGATTTGCACCAGAAC	GU722160	202	78.50
RC24	F-TCTGCTGCAGCCAGTAC<br>R-GGAGAAGCATCTGGTCGAAG	X87109.1	174	79.00
OsPR5	F-GCCACTTCTTCCGCTATCCT<br>R -CACGTTGATGGTCCACGTAT	X68179	174	81.50
OsAOS2	F-AAGAAGGGGGAGATGCTGTT<br>R-TGCTTGTTGTCAACGCTAGG	AY055775.1	182	79.50
OsMAPK6	F-TATGTGCACTCGGCAAATGT<br>R-TCGATACCAACGAGTGACCA	AB183398	156	82.00
OsPR10a	F- AGCTGGCCATTGCTTTGG<br>R-GCTTCGTCTCCGTCGAGTGT	AF274850	65	79.00
OsPR1b	F-AGCTGGCCATTGCTTTGG<br>R-CGTTGTGGAGCCTCACGTAGT	EF061247	90	80.50
β-tubulin	F-GGAGTCACATGCTGCCTAAGGTT<br>R-TCACTGCCAGCTTACGGAGG	X78143	64	77.50

Supplementary Table.3 Relative fold change of endogenous *PR* marker, defense related, JA and SA pathway genes in qRT-PCR after 24 and 48hr post infection

	Relative fold change															
	OsPR5		*OsMAPK6*		*OsNH1*		*OsPR10a*		*OsPR1b*		*OsAOS2*		*OsPAL*		*RC24*	
Transgenic lines	24h	48h	24h	48h	24h	48h	24h	48h	24h	48h	24h	48h	24h	48 h	24h	48h
CN5-2-1	1.15	0.90	0.92	1.05	8.46	3.87	3.34	2.78	1.21	2.17	1.57	3.23	1.14	1.36	0.73	3.63
C8-9-1	0.54	0.64	0.44	0.65	3.46	2.04	2.52	2.06	0.65	1.04	1.71	2.37	0.60	0.77	0.28	2.41
N4-3-2	0.78	0.64	0.51	1.01	4.96	2.48	2.18	2.34	0.92	1.87	1.57	3.25	1.49	1.85	0.37	1.63
